# Construction of Fluorescent Immunosensor Quenchbody to Detect His-Tagged Recombinant Proteins Produced in Bioprocess

**DOI:** 10.3390/s21154993

**Published:** 2021-07-22

**Authors:** Xuerao Ning, Takanobu Yasuda, Tetsuya Kitaguchi, Hiroshi Ueda

**Affiliations:** 1 Tokyo Institute of Technology, Graduate School of Life Science and Technology, 4259-R1-18 Nagatsuta-cho, Midori-ku, Yokohama 226-8503, Kanagawa, Japan; neisan@pe.res.titech.ac.jp (X.N.); yasuda.t.af@m.titech.ac.jp (T.Y.); 2 Laboratory for Chemistry and Life Science, Institute of Innovative Research, Tokyo Institute of Technology, 4259-R1-18 Nagatsuta-cho, Midori-ku, Yokohama 226-8503, Kanagawa, Japan; kitaguchi@pe.res.titech.ac.jp

**Keywords:** His-tag, fluorescent biosensor, immunoassay, recombinant protein production

## Abstract

With the widespread application of recombinant DNA technology, many useful substances are produced by bioprocesses. For the monitoring of the recombinant protein production process, most of the existing technologies are those for the culture environment (pH, O_2_, etc.). However, the production status of the target protein can only be known after the subsequent separation and purification process. To speed up the monitoring of the production process and screening of the higher-yield target protein variants, here we developed an antibody-based His-tag sensor Quenchbody (Q-body), which can quickly detect the C-terminally His-tagged recombinant protein produced in the culture medium. Compared with single-chain Fv-based Q-body having one dye, the Fab-based Q-body having two dyes showed a higher response. In addition, not only was fluorescence response improved but also detection sensitivity by the mutations of tyrosine to tryptophan in the heavy chain CDR region. Moreover, the effect of the mutations on antigen-binding was successfully validated by molecular docking simulation by CDOCKER. Finally, the constructed Q-body was successfully applied to monitor the amount of anti-SARS CoV-2 nanobody secreted into the *Brevibacillus* culture media.

## 1. Introduction

Recently, with the gradual maturity of DNA recombination technology, an increasing number of useful substances are produced in the form of recombinants through bioprocesses, which overcomes the limitation of extraction from natural sources. The monitoring and control of this process are very important for obtaining high-quality products. For this reason, people have developed a variety of sensor systems. However, most of these sensor technologies target environmental variables, such as temperature, dissolved oxygen/carbon dioxide, and metabolites [1]. To analyze target products, the subsequent purification and concentration steps are usually needed. This undoubtedly consumes a lot of labor and time for the screening of productive and high-quality products with many variables that need to be optimized [2]. Therefore, a sensor that can directly measure the target product in bioprocesses is still in demand for analysis of the expected substance in the screening process and high-efficiency monitoring in large-scale production.

Among the recombinant proteins produced by bioprocesses, most of them have a His tag [3]. This is a fusion tag designed to purify the desired protein by an immobilized metal-affinity column (IMAC), so that people can obtain products with high purity. In this circumstance, people have become increasingly aware of the importance of specific detection of His-tagged recombinant protein. At present, a number of detection methods for His-tagged proteins have been developed. Based on traditional immunoassay methods, such as Western blotting and enzyme-linked immunosorbent assay (ELISA), which require at least 4~5 h of operating procedure, an emerging UVHis-PAGE method was developed [4]. It simplifies the blotting step after PAGE and eliminates the expensive instruments and consumables required for detection, but this method still needs to run SDS-PAGE followed by at least 1 h of incubation and rinse steps. Another example is the lateral flow (LF)-based test strip on the market, which can detect His-tagged protein at the concentration range of 4–20 μg/mL within 15 min (“Pro-Detect Rapid assays”, Thermo Fisher Scientific Co., Waltham, MA, USA). However, this still suffers from a high detection limit and narrow detection range. Although the latest study declared that faster detection (10 min) with a densitometric detection limit of 0.5 μM has been realized, the method is still difficult for carrying out accurate quantification [5]. Also, by combining DNA aptamer and gold nanoparticle technologies, quick and qualitative determination of His-tagged protein was reported. However, their working range is narrow [6,7]. This is not conducive to knowing when to harvest the target recombinant products in large-scale production, which may result in a decreased yield due to premature recovery or protein degradation. On the other hand, a kind of fluorescent probe for His-tagged proteins has been developed [8]. The assay based on this probe is fast (90 s) and has the detection limit of 0.8 μM for His_6_-proteins, but it requires an additional reagent, a peptide that acts as the donor for phosphorescence detection. This means that multiple components are necessary to perform competitive measurement, which is not ideal as a biosensor. Therefore, to cater to the rapidly changing bioprocess, especially the cases in which a microorganism is used as an expression host, a more simplified and rapid His-tagged protein monitoring method is still expected.

Previously, we developed a fluorescent antibody probe Quenchbody (Q-body) [9,10]. This is generated by certain fluorescent dyes such as tetramethylrhodamine (TAMRA) specifically labeled near the antigen-binding site of the antibody. Under the effect of tryptophan (Trp) residues inside the antibody, the fluorescent dye can be quenched by a photo-induced electron transfer (PeT) mechanism. Upon the binding of antigen to the antibody, this quenching effect is disturbed, so that the dye regains its fluorescence signal. The Q-body method can specifically detect various antigen molecules rapidly, and the operation is very simple. Just by adding Q-body into the sample containing the analyte and simply mixing without extra reagents, the fluorescence signal can be detected immediately. Because of these advantages, Q-body made of many formats of antibodies has been used as a convenient and fast biosensor targeting many useful substances, such as osteocalcin (BGP) and methotrexate (MTX) [11,12].

This time, with the aim of easier monitoring of secreted His-tagged recombinant proteins during bioprocesses, we tried to develop a Q-body that can quickly and specifically recognize recombinant proteins with a C-terminal His tag in the culture environment. In this study, we used anti-C-terminal His tag antibody 3D5 (PDB 1KTR) [13] labeled with maleimide fluorescent dyes at the N-terminal region of its heavy and light chains to construct a Q-body that shows sufficient fluorescence quenching and antigen-dependent de-quenching. In addition, we investigated the effect of adding Trp residues at the antigen-binding site and successfully improved not only the fluorescence response but also the antigen-binding affinity and detection sensitivity of the Q-body. Finally, the constructed Q-body was applied to monitor the amount of the His-tagged nanobody protein secreted into the *Brevibacillus* culture media. *Brevibacillus* is a Gram-positive bacterium known for its ability in secretive expression of exogeneous proteins, and does not secrete proteases [14] that can cause degradation of the secreted proteins in the culture media. The production and use of the V_HH_ nanobody, which is derived from a Camelid single chain antibody, are becoming increasingly popular due to its small size and superior stability [15,16,17]. We tried to monitor the expression of anti-SARS-CoV-2 spike V_HH_ nanobody from the cultured *Brevibacillus* cells.

## 2. Materials and Methods

### 2.1. Materials

Restriction endonuclease was from New England Biolabs Japan (Tokyo, Japan). The In-Fusion HD cloning kit, pGro7 chaperonine plasmid, Talon metal affinity resin, Talon disposable gravity column, and a *Brevibacillus* In Vivo Cloning (BIC) system were from Takara Bio (Otsu, Japan). The KOD Plus mutagenesis kit was from Toyobo Biochemicals (Osaka, Japan). The PureYield Plasmid Miniprep System and Wizard SV Gel and PCR Clean-Up System were from Promega (Tokyo, Japan). MicroSpin G-25 Columns were from GE Healthcare (Amersham, UK). The Luria-Bertani medium was from Beckton-Dickinson (Tokyo, Japan). Immunoblock was from KAC (Hyogo, Japan). 5-TAMRA C6 maleimide was from AAT Bioquest (Sunnyvale, CA, USA). Oligonucleotides and the gene for 3D5 scFv were synthesized by Eurofins Genomics (Tokyo, Japan). The sequence of primers used is summarized in Table S1. Ultrapure water was prepared using a Milli-Q machine (Millipore Japan, Tokyo, Japan). Biotinylated His_6_ peptide was synthesized using a PSSM-8 peptide synthesizer (Shimadzu, Kyoto, Japan). Other chemicals and reagents, unless otherwise indicated, were from Sigma-Aldrich (St. Louis, MO, USA) or Fujifilm-Wako pure chemicals (Osaka, Japan).

### 2.2. Construction of scFv and Fab Expression Plasmids

A single-chain Fv (scFv) expression vector pSQ (KTM219) [18] encoding a Cys-tag containing a cysteine (Cys) residue, anti-osteocalcin scFv, a His_6_ (HHHHHH)- and a FLAG (DYKDDDDK)- tag at its C- terminal was digested by *Age*I-HF and *Xho*I-HF to remove the original scFv fragment and ligated with *Age*I-HF- and *Sal*I-HF-digested V_H_-V_L_ type 3D5 scFv fragment in pEX-K4J2-1KTR, which was amplified by primers 3D5_Age_back and 3D5_Sal_for, and KOD-plus Neo DNA polymerase.

An antigen-binding fragment (Fab) expression vector pUQ2(KTM219) encoding two Cys-tags, each containing a Cys residue at the N-terminus of both V_H_ and V_L_ was appended with a His_6_ -myc tag at the C- terminus of the heavy chain and a FLAG tag at the C- terminus of the light chain. This plasmid was firstly digested by *Age*I-HF and *Xho*I-HF to remove the original VH fragment and in-fused with a 3D5 VH fragment, which was amplified using the primers Age_back and In-fusion_3D5_VH_for and pEX-K4J2-1KTR encoding (GGGS)_4_-V_H_-V_L_ 3D5 scFv as a template, using KOD-plus neo DNA polymerase. After that, it was digested by *Spe*I-HF and *Hin*dIII-HF to remove the original VL fragment and in-fused with the 3D5 VL fragment, which was amplified from the same template by primer In-fusion3D5VLback and In-fusion3D5VLfor. The vector thus made was digested with *Age*I-HF to remove the (GGGS)_4_ linker between Cys-tag and VH to make a linker-less pUQ2 (3D5).

To introduce Y32W and/or Y33W mutations in the V_H_ region of scFv and Fab, a short DNA fragment was amplified by a primer pair of 3D5_Age_back and 3D5VH31for, and a longer DNA fragment was amplified by another primer pair of either 3D5VH_WY_back, 3D5VH_WW_back or 3D5VH_YW_back and 3D5_Sal_for. After running agarose electrophoresis (1.5% agarose), the bands of 164 bp and 701 bp were recovered. Overlap extension PCR was performed on these DNA fragments, and the product was amplified using VHH_Age_back and 3D5_Sal_for. After being digested by *Age*I-HF and *Xho*I-HF, the 3D5_WY/WW/YW_V_H_ insert was ligated with pSQ (3D5) or pUQ2 (3D5) digested with the same enzymes using ligation high ver2 enzyme, to yield WY/WW/YW-mutated pSQ (3D5) and pUQ2 (3D5), respectively.

### 2.3. Protein Expression and Purification

SHuffle T7 Express *E. coli* cells were co-transformed with pGro7 chaperone plasmid and one of pSQ/pUQ2 (3D5) plasmid and cultured at 30 °C overnight on LBAC (Luria-Bertani medium containing 50 μg/mL ampicillin and 20 μg/mL chloramphenicol) plate containing 1.5% agar. A colony was picked and cultured at 30 °C, 200 rpm in 4 mL LBAC medium for one night. The next day, transferred 4 mL bacterial culture into 400 mL LBAC medium and cultured at 30 °C, 200 rpm until OD_600_ reached 0.4~0.6. After 0.4 mM isopropyl β-d-1-thiogalactopyranoside induction, the bacterial solution was incubated at 16 °C, 200 rpm for 16 h, and centrifuged at 4 °C, 5000 g for 15 min. The *E. coli* cells were resuspended with 9 mL of extraction buffer (20 mM Na_2_HPO_4_, 20 mM NaH_2_PO_4_, 500 mM NaCl, pH 7.4) and disrupted by a cell disruptor One Shot Model (Constant Systems, Daventry, UK). After being centrifuged at 4 °C, 8000× *g* for 10 min, the supernatant was incubated with 200 μL Talon IMAC resin on a rotating wheel at 4 °C for 1 h. After centrifugation at 4 °C, 700× *g* for 1 min, the resin was transferred to a 2 mL disposable gravity column. The resin was washed with 500 μL of binding buffer (20 mM Na_2_HPO_4_, 20 mM NaH_2_PO_4_, 500 mM NaCl, 20 mM imidazole, pH 7.4) 3 times, and the target protein was eluted with 400 μL elution buffer (10 mM Na_2_HPO_4_, 10 mM NaH_2_PO_4_, 250 mM NaCl, 500 mM imidazole, pH 7.4). The yield and purity of the target protein were confirmed by SDS-PAGE, which was loaded with the same volume of 2 × SDS loading buffer (125 mM Tris-HCl, 4% SDS, 20% glycerol, 0.02% bromophenol blue, 0.2 M dithiothreitol, pH 6.8), and boiled at 95 °C for 5 min. The protein concentration was determined by using a densitometer WSE-6100 and a CS Analyzer 4 software (ATTO, Tokyo, Japan) by comparing it with several concentrations of bovine serum albumin (BSA) as a standard.

### 2.4. Enzyme-Linked Immunosorbent Assay

Each well of a Costar 3590 96-well microplate (Corning, Tokyo, Japan) was coated with or without 100 μL of thioredoxin (Trx)-fused LnBiT-His protein (2 μg/mL, His_6_ tag at the C-terminus) [19] in PBS and incubated at 4 °C overnight. The plate was washed 3 times with 200 μL PBS containing 0.1% Tween20 (PBST) and blocked with 200 μL of PBST containing 20% Immunoblock (KAC, Japan) at 25 °C for 1 h. After the plate was washed three times with PBST, 100 μL of anti-His scFv/Fab 3D5 (2 μg/mL) in PBST containing 5% Immunoblock was added, and then the plate was incubated at room temperature for 2 h. After washing the plate three times, bound scFv/Fab 3D5 was probed with 100 μL of 1:2000 diluted horseradish peroxidase (HRP)-labeled anti-FLAG M2 (Sigma) in PBST containing 5% Immunoblock at 25 °C for 1 h. After it was washed three times with PBST, 100 μL of substrate solution (100 mM CH_3_COONa, 0.2 mg/mL TMBZ, 0.09% H_2_O_2_, pH 6.0) was added, and 50 μL of stop solution (10% H_2_SO_4_) was added when the solution turned blue. The absorbance at 450 nm with a reference at 655 nm was monitored by a microplate reader SH-1000 (Corona Electric, Ibaraki, Japan).

### 2.5. Fluorescence Labeling and Purification

The purified protein (10 μM) in 120 μL PBST was mixed with 8 mM tris (2-carboxyethyl) phosphine (TCEP)-HCl in a 1.5 mL tube for 20 min at 25 °C. To inactivate TCEP, 20 mM 4-azidobenzoic acid (ABA), pH 7.0, was added [20], and the tube was put in a vacuum for 15 min to remove air bubbles. Afterwards, 20 × mol of 5-TAMRA C6 maleimide or ATTO520-C2 maleimide in DMSO was added and it was incubated at 4 °C for 16 h or 25 °C for 1 h. Anti-FLAG M2 magnetic beads (Sigma, 10 μL) were washed with PBST, and then the beads were added to the reaction mixture. After incubation on a rotating wheel at 25 °C for 1 h, the beads were washed 12 times with PBS containing 0.1% Brij35 and 2 times with PBST. Then 100 μL of 150 μg/mL 3×FLAG peptide in PBST was used to elute the labeled 3D5 Q-body. The 3×FLAG peptide was removed by using a MicroSpin G-25 Column (GE Healthcare, Amersham, UK), which was pre-equilibrated with PBST.

### 2.6. Fluorescence Measurements

The Q-body sample solution (100 μL) (n = 3) was applied into a well of black 96-well half area microplate (Greiner Bio-One GmbH, Frickenhausen, Germany). The fluorescence measurement was carried out immediately on a CLARIOstar plate reader (BMG Labtech, Ortenberg, Germany). The wavelengths of 535/20 nm and 585/30 nm for TAMRA, and 490/20 nm and 550/40 nm for ATTO520 were used for the excitation and emission, respectively. In each measurement, the fluorescence background of the sample solution without Q-body was subtracted. To derive EC50 and limit of detection (LOD), antigen dose-response curves were fitted to a four-parameter logistic equation using Kaleidagraph 4.5 (Synergy Software, Reading, Mount Penn, PA, USA) as follows:

y = d + (a − d)/(1 + (x/c)^b^) (1)

where a was set to 1. EC50 was taken from c, and LOD calculated as the concentration corresponding to the mean blank value plus three times the standard deviation for each assay.

### 2.7. Antigen-Binding Kinetics Analysis

To evaluate the antigen-binding affinity of the proteins, bio-layer interferometry (BLI) measurements were performed on an Octet K2 system (Pall FortéBio, Fremont, CA, USA). Streptavidin-conjugated (SA) biosensors were soaked in Kinetic buffer (10 mM Na_2_HPO_4_, 10 mM NaH_2_PO_4_, 150 mM NaCl, 0.002% Tween20, 0.1% BSA, pH 7.4) for 10 min. After that, 100 nM biotinylated His_6_ peptide (bio-EGGGSHHHHHH-COOH, synthesized as in Figure S1, Supplementary materials) was loaded on an SA biosensor and equilibrated in Kinetic buffer before analysis. For each measurement, 80 μL of unlabeled protein or Q-body in Kinetic buffer in two-fold gradient concentrations were applied, which was followed by the cycle of: 1000 rpm shake speed, baseline measurement in Kinetic buffer for 1 min, association measurement in a sample for 200~400 s, and dissociation measurement in Kinetic Buffer for 200~400 s. The biosensors were regenerated in 500 mM phosphoric acid (pH 1.0) for 1 min and equilibrated with Kinetic buffer for 30 s before the next cycle. The data were imported into Data Acquisition 11.0 (FortéBio) and the kinetic constants were calculated by Data Analysis 11.0 (FortéBio) using double reference subtraction assuming a 1:1 binding model.

### 2.8. Secretive Expression and Analysis of His-Tagged Nanobody

The anti-SARS-CoV2 VHH gene tagged with a hinge, 3 × FLAG and C-terminal His tags (Kindly provided by Dr. Akikazu Murakami in RePhagen Inc., Okinawa, Japan) was PCR amplified with primers pBIC_pET1-4VHH_Rv and pBIC3-forwardVHH, and co-transformed to *Brevibacillus choshinensis* HPD31 cells with pBIC3 vector (Takara Bio) so that the plasmid encoding target gene with a C-terminal His tag was formed by homologous recombination in *Brevibacillus* cells. The cells were spread on an MTNm agar plate (TM medium, which is 10 g/L glucose, 10 g/L phytone peptone, 5 g/L Ehrlich bonito extract, 2 g/L yeast extract, 10 mg/L FeSO_4_·7H_2_O, 10 mg/L MnSO_4_·4H_2_O, 1 mg/L ZnSO_4_·7H_2_O, pH 7.0, containing 50 μg/mL neomycin, 20 mM MgCl_2_, and 1.5% agar), cultured at 37 °C for 16 h and colony PCR was performed to confirm the presence of the insert DNA.

For the analysis of V_HH_ secretion level in M9 medium (1 g/L NH_4_Cl, 3 g/L KH_2_PO_4_, 6 g/L Na_2_HPO_4_·7H_2_O, 1 mM MgSO_4_, 0.00005% Vitamin B1, 0.1% casamino acid, 1% glucose), a single colony was selected and pre-cultured in 4 mL TMNm (TM medium containing 50 μg/mL neomycin) medium at 30 °C, 150 rpm for 48 h. The cultured bacterial solution was centrifuged at 22 °C, 5000 rpm for 5 min. After it was washed by M9Nm (M9 medium containing 50 μg/mL neomycin) medium, the pellets were cultured in 4 mL M9Nm medium 30 °C, 150 rpm. For the analysis of V_HH_ production by the Q-body assay, a single colony was inoculated in a 4 mL TMNm medium and cultured at 30 °C, 150 rpm for one night. The next day, 2 mL of the culture was inoculated into 100 mL TMNm medium to continue the culture at 30 °C, 150 rpm, until OD_600_ = 2. After the bacterial culture was centrifuged at 22 °C, 5000 rpm for 5 min, the pellets were washed with M9Nm medium and cultured in 100 mL M9Nm medium at 30 °C, 120 rpm. The culture time mentioned in this study was all counted from the exchange of M9Nm medium. The culture media at several time points were collected and centrifuged under the same condition as above. The supernatant was diluted with the same volume of PBST, and the V_HH_ secretion level was analyzed by the Q-body assay and SDS-PAGE and Coomassie Brilliant Blue (CBB) staining.

### 2.9. Docking Analysis

A docking simulation based on the PDB structure 1KTR with bound ligand (His_4_) was performed using CDOCKER, a grid-based molecular dynamics docking algorithm [21], in Discovery Studio v18.1.0.1 (DS, Dassault Systèmes BIOVIA, Vélizy-Villacoublay, France). First, the “Prepare Protein” command in DS was used to automatically correct structural problems such as missing linker sequences and missing amino acid residues in the scFv. Then, the energy of the complex structure was minimized in 200 steps using the smart minimize algorithm. After that, a site sphere with a radius of 10.3 Å was built based on the ligand of the complex structure, before the His_4_ ligand was removed from the model. The docking simulation was carried out using the remaining ligand. CHARMm was selected as the force field, the heating steps were set to 2000 and the heating target temperature was set to 700, the cooling steps were set to 5000 and the cooling target temperature was set to 300. The 10 generated docking poses were then ranked based on their -CDOCKER Energy and -CDOCKER Interaction Energy. -CDOCKER Energy and -CDOCKER Interaction Energy were used as indicators for the quality of molecular docking. The high positive value of these indicators presented a good interaction between the ligand and the receptor. The poses with the highest -CDOCKER Energy and -CDOCKER Interaction Energy were selected as the best poses for further analysis. 1KTR with a modified structure was mutated to minimize the energy for each mutant, and docking simulation was performed using the same procedure as for the wild-type.

## 3. Results and Discussion

### 3.1. Preparation of scFv/Fab 3D5 and Their Variants

**Figure 1:**
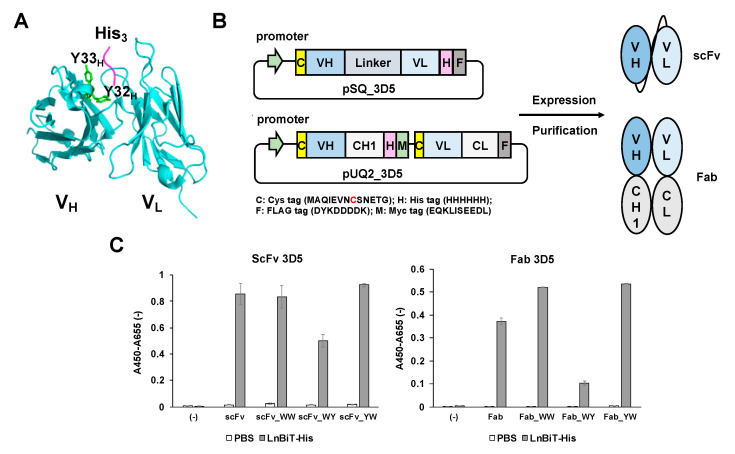
Protein expression and activity of anti-His scFv/Fab 3D5. (**A**) Structure of 3D5 Fv complexed with His6. (**B**) Scheme of the plasmids construction and protein expression for scFv and Fab 3D5 fragments (Figure S1). (**C**) Specific antigen binding of scFv/Fab 3D5 fragments and their variants detected by enzyme-linked immunosorbent assay (ELISA). Gray bars represent the signals with immobilized thioredoxin-fused LnBiT protein with His6 tag at the C-terminus. White bars represent the signals without antigen. Error bars indicate ±1 standard deviation (SD) (n = 3).

First, we made expression plasmids for scFv and Fab fragments, having one or two N-terminal Cys tag(s) at their N-terminus, respectively. The plasmid for scFv 3D5 encodes an N-terminal Cys tag, scFv, and a C-terminally located His- and Flag- tags. The plasmid for Fab 3D5 included a Cys tag at the N-terminus of both heavy chain and light chain, and a His-tag at the C-terminus of the heavy chain, followed by a myc tag, which prevented self-recognition of the 3D5 antibody (Figure 1A,B).

Since the preliminary experiment of scFv-based Q-body showed limited quenching behavior, we reasoned that the number of Trp residues in 3D5 Fv is not enough, especially with no Trp in the complementarity determining region (CDR). Hence, in addition to scFv and Fab fragments having the original 3D5 sequence (here we refer to this as the wild-type), we also made three types (WW, YW, and WY) of mutants with the mutations at the antigen binding site in the V_H_ to increase the degree of quenching. We chose two tyrosine (Tyr) residues Y32_H_ and Y33_H_ in CDR_H1_, according to the Kabat numbering scheme [22]. In particular, Y33_H_ was in close proximity to the bound His residue of the antigen (Figure 1A and Figure S2). We reasoned that changing these non-quenching Tyr residues to Trp with similar structure will increase the quenching while maintaining the antigen binding affinity. The position of Trp and selected Tyr residues in CDR_H1_ are shown in Table S2.

The scFv or Fab 3D5 fragment was co-expressed in *E. coli* having oxidized cytoplasm with a chaperonine GroES-GroEL encoded in the plasmid pGro7 [23,24], which was found to increase the soluble expression of 3D5 antibody fragments. The Fv and Fab were purified via internal His-tag using an immobilized metal affinity column. After running the products on a 10% SDS-PAGE gel and stained with CBB, the bands around 30 kDa in each lane were observed, which corresponded well with the theoretical molecular weights of scFv and Fab (Figure S3; scFv: 29.5 kDa, heavy chain of Fab (Fd): 27.1 kDa, Light chain (Lc): 26.2 kDa). Although some additional bands including that for GroEL appeared, this result proved that all of the scFv and the two chains of Fab 3D5 fragments were obtained with similar amounts, with the yields of ~0.15 mg of scFv and ~0.4 mg Fab per 1 L culture, respectively.

The antigen-binding activity of each recombinant 3D5 was determined by ELISA, with immobilized Trx-LnBiT (His6 tag at the C-terminus) as an antigen, and the bound scFv/Fab were detected by anti-FLAG antibody. For scFv and Fab, WW and YW variants exhibited the same or higher antigen-binding activity compared with the wild type (Figure 1C), while the WY variants of both scFv and Fab showed decreased absorbance signals. This suggests that WW and YW mutations made a positive contribution to the antigen-binding activity of 3D5 antibody fragments.

### 3.2. Construction of Anti-His Tag Q-Bodies

**Figure 2:**
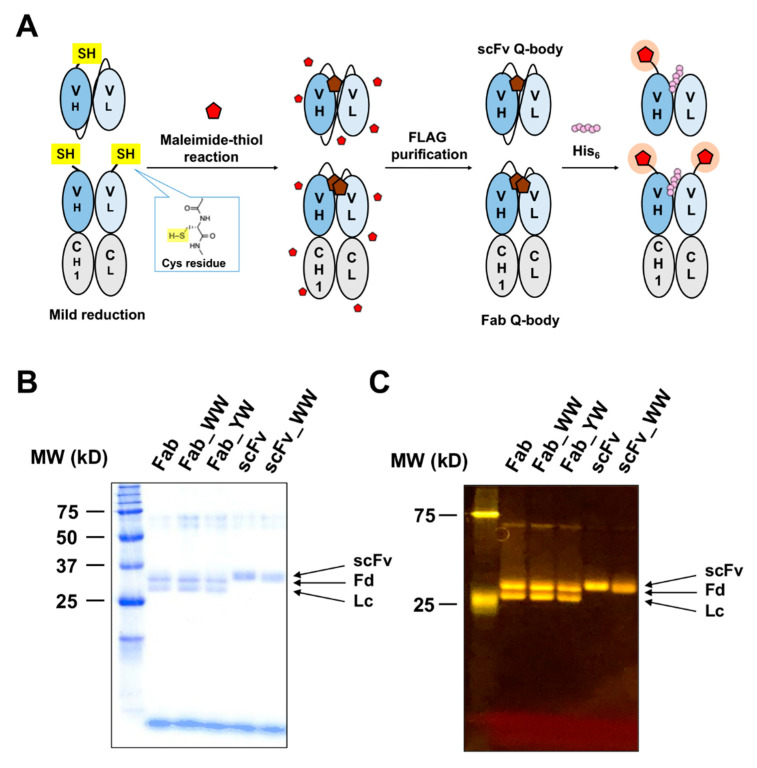
Construction of scFv/Fab Q-bodies labeled with maleimide dye at the N-terminus. (**A**) Scheme for the construction of scFv- and Fab-type Q-bodies labeled with maleimide dye at the N-terminus of H chain in scFv, and at both heavy (Fd = V_H_-C_H_1) and light (Lc = V_L_-C_L_) chains in the Fab. (**B**) Coomassie Brilliant Blue (CBB)-stained and (**C**) fluorescence images of 5-TAMRA (tetramethylrhodamine) C6 maleimide-labeled wild-type scFv/Fab Q-body and their variants.

Previously, because many antibody fragments labeled with 5-TAMRA C6 maleimide dye exhibited desirable Q-body performance, the purified scFv and Fab fragments of 3D5 were labeled with the TAMRA dye at the N-terminal Cys tag through maleimide-thiol reaction. After removing excess dye by FLAG-tag purification and G25 desalting column (Figure 2A), the proteins were analyzed by SDS-PAGE. The images of CBB-stained and fluorescence SDS-PAGE proved that these Q-bodies have been successfully labeled with fluorescent dyes (Figure 2B).

**Figure 3:**
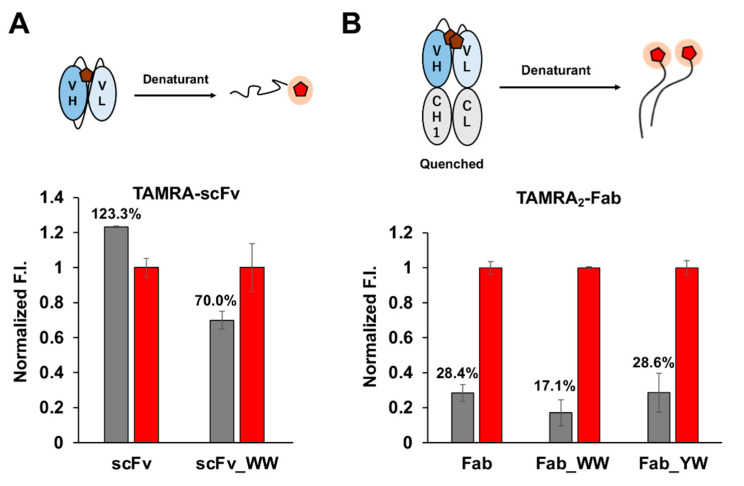
Quenching efficiency of 5-TAMRA C6 labeled antibody fragments, where the fluorescence intensity of denatured state was set to 1. (**A**) Quenching efficiency of single labeled scFv fragment with and without WW mutation, and (**B**) that of double-labeled Fab Q-bodies. Gray and red bars represent the fluorescence intensity of quenched and denatured Q-bodies, respectively. The fluorescence intensities were normalized by the mean intensity of each Q-body (1 nM) in the denaturant. Error bars indicate ±1 standard deviation (SD) (n = 3).

Hence, the quenching efficiency of Q-bodies, which is a good indicator of the antigen-dependent fluorescence response due to de-quenching, was measured in the presence of the denaturant (7 M guanidine hydrochloride, 100 mM dithiothreitol) using a fluorescence plate reader. For scFv, only the WW-mutated Q-body showed some quenching effect (Figure 3A), while the others did not (Figure S4). Moreover, in the presence of His_6_ peptide at the concentrations of 30 μM and 100 μM, the de-quenched fluorescence signal displayed by TAMRA-labeled WW_scFv Q-body was higher than that of the wild-type scFv Q-body, even though the signals were not very significant (less than 2-fold, Figure S4). However, the increase of quenching efficiency caused by the introduction of mutations was noteworthy (130.1% became 70.0% for WW, 94.3% for WY, and 131.1% for YW).

As for the double-labeled Fab Q-bodies, all the wild-type and mutant clones displayed significant quenching (Figure 3B). Among them, WW-mutant Fab displayed better quenching (to 17.1%) than YW- and the wild-type Fab (28.6% and 28.4%, respectively). Therefore, the evaluated quenching effect of Q-bodies by denaturant is considered credible. These results indicated that double-labeled Fab could achieve deeper quenching than single-labeled scFv. This phenomenon revealed that the quenching of fluorescence dye is not only due to the electron transfer from the internal Trp residues, but also benefits from the dye-dye quenching to a large extent. In addition, WW-mutation was found to be the most effective mutation as for the quenching in both scFv and Fab.

### 3.3. Binding Kinetics of scFv/Fab 3D5 and Their Q-Bodies

To understand the antigen-binding activity of anti-His scFv/Fab fragments and their Q-body derivatives more clearly, we utilized bio-layer interferometry to study the binding kinetics and binding affinity of 3D5 antibodies and their Q-bodies to biotinylated His_6_ peptide immobilized on the surface of a streptavidin biosensor. The sensorgrams were fitted globally to obtain the association rate constant (ka), the dissociation rate constant (kd) and the equilibrium dissociation constant (K_D_) assuming a 1:1 binding model (Table 1). The raw sensorgrams are shown in Figure S5.

As a result, scFv 3D5 and Fab 3D5 bound to His_6_ peptide with low equilibrium dissociation constants of 5.0 nM and 3.6 nM, respectively. The reason why Fab has higher affinity than scFv might be due to more stable structure of Fab, which is similar to that of whole IgG. It is also worth noting that the WW and YW mutants of scFv and Fab exhibited higher affinity to His_6_ than their wild-type counterparts. Interestingly, before dye-labeling, the affinity of Fab_WW mutant was stronger than the YW mutant or the wild-type Fab. This means that the Y to W mutations, especially at 33H were indeed effective in raising the antigen-binding affinity. However, when the fragments were labeled with TAMRA, the affinity of WW mutant got considerably decreased, compared with others (scFv_WW: 2.88 nM → too high; scFv: 5.03 → 26.8 nM) (Fab_WW: 0.17 → 3.19 nM; Fab_YW: 0.76 → 1.31 nM; Fab: 3.62 → 3.19 nM). These results clearly show that the labeled TAMRA dye(s) competes with the antigen binding, and the more Trp residues exist in the CDR, the more competition is induced. This result probably reflects the strong π–π interaction between the stacked Trp and TAMRA (see Section 3.6).

In summary, the results obtained illustrate that WW/YW mutation in the Fab V_H_ region improved the affinity of Fab to His_6_ antigen, and this was also true for the Fab Q-bodies. It is worth noting that while scFv WW Q-body showed no detectable binding, it showed some antigen-dependent fluorescence (Figure S4B). Hence, the affinity of this Q-body became lower than the detection limit of BLI after TAMRA labeling.

### 3.4. His6 Peptide-Dependent Fluorescence Response of Fab Q-Bodies

**Figure 4:**
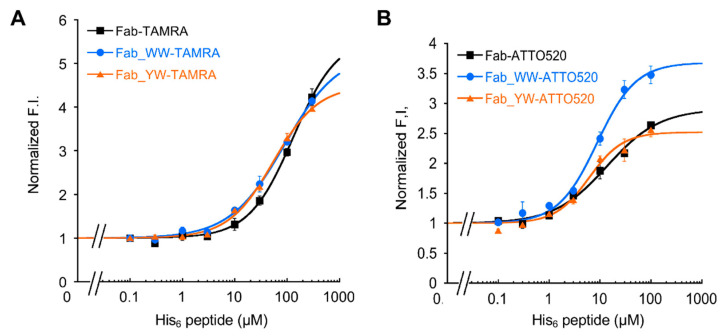
His_6_ peptide dose-response of the wild-type and mutant Fab Q-bodies. His_6_ dose-response curves of 5-TAMRA C6- (**A**) or ATTO520-C2 (**B**) labeled Fab-type Q-body in PBST. Error bars indicate ±1 SD (n = 3).

Because of the significant quenching effect and the high affinity of Fab Q-bodies, the antigen-dependent fluorescence response of the two Fab-type Q-bodies was further investigated. In addition to the labeling with 5-TAMRA C6 maleimide, labeling with another preferred dye ATTO520-C2 maleimide, which has a similar chromophore to that of TAMRA, was also tried. With varied concentrations of His_6_ peptide as an antigen, the fluorescence signals from these Q-bodies were measured. For TAMRA-labeled Q-bodies, the standard curves of all three Fab-type Q-bodies displayed a notable increase in fluorescence intensity as His_6_ peptide increased (Figure 4A). Through the curve fitting, the EC50 values of WW- and YW-TAMRA Q-bodies were calculated as 89.5 μM and 54.7 μM, respectively. It is of note that these values were lower than that of the wild-type TAMRA Fab Q-body (EC50: 132 μM), besides their LOD values being similar (Table 2). On the other hand, ATTO520-labeled Q-bodies generally showed higher sensitivity than TAMRA-labeled ones (Figure 4B). WW- and YW- Q-body variants showed an even higher sensitivity for His_6_ peptide than the wild-type Q-body.

Meanwhile, the BLI results showed a higher affinity of these Q-bodies to His_6_ peptide, with the K_D_ values below 30 nM. However, their μM range EC50 values were quite different from their K_D_ values. This might be due to the mutual quenching effect called H-dimer formation between two rhodamine dyes [15], and TAMRA-TAMRA quenching is stronger than that between ATTO520, which explains why the maximum upper limit of de-quenching of TAMRA is higher than that of ATTO520. However, the small size of the His_6_ peptide made it difficult to interfere with the strong dye-dye H-dimer stacking to fully release the fluorescence signal, and this might lead to the higher EC50 of Q-body response than its K_D_ value. In summary, the improved detection sensitivity of Fab Q-body variants indicates that WW and YW mutation in the V_H_ region caused positive effects to convert Fab to Q-body.

### 3.5. Application of Q-Body to Quantify His-Tagged Protein Produced in Bioprocess

**Figure 5:**
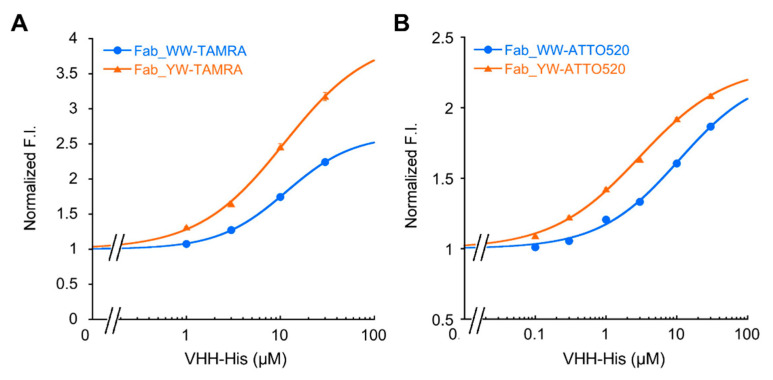
Responses of Q-bodies to His-tagged protein. Dose-response curve for spiked C-terminal His6-tagged nanobody detected by TAMRA- (**A**) and ATTO520- (**B**) labeled Fab-type Q-bodies in 50% M9 culture medium after cultivation with *Brevibacillus choshinensis* HPD31. Error bars indicate ±1 SD (n = 3).

Because Fab Q-bodies displayed significant dye-quenching and antigen-dependent fluorescence response, we tested the possibility of its application to an actual bioprocess. To this end, we made a secretory expression system of a nanobody that binds the receptor binding domain of SARS-CoV-2 S protein, based on *Brevibacillus choshinensis* HPD31 (Figure S5A). We did not employ *E. coli* secretion-expression system because of its low yield. We measured the fluorescence signal of Q-body to different concentrations of spiked C-terminally His-tagged V_HH_ nanobody in 50% M9 culture medium of *Brevibacillus*. We also expected that with the use of larger protein antigen rather than small peptide, higher interference with the dye-dye interaction might be observed when bound to a Fab Q-body, and results in higher fluorescence dequenching. As a result, both TAMRA- and ATTO520-labeled Fab Q-body variants were found to work in the presence of 50% culture medium (Figure 5).

Another finding was that, although the buffer condition was somewhat different, as expected, compared with His_6_ peptide detection, the Q-bodies in 50% M9 all showed improved detection sensitivity to the His-tagged nanobody in terms of EC50 and LOD (Table 3). This may be because the size of the V_HH_-His protein is larger than that of the His_6_ peptide, and it is easier to spatially interfere with the dye-dye quenching when it enters the antigen-binding pocket between V_H_ and V_L_.

**Figure 6:**
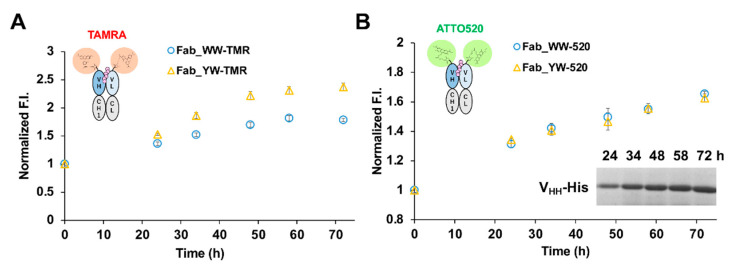
Responses of Q-bodies to His-tagged protein. His_6_-tagged V_HH_ secreted by the transformed Brevibacillus in M9 culture was detected by TAMRA- (**A**) and ATTO520-(**B**) labeled Fab-type Q-bodies in 50% M9 culture medium. Error bars indicate ±1 SD (n = 3). Inset: CBB stained gel of V_HH_-His protein in the culture medium at indicated time points.

Next, we used the Q-bodies to determine the amount of V_HH_-His protein secreted by *Brevibacillus* in 50% M9 culture medium at different time points. As the culture time passes, the fluorescence signal displayed by the Q-body became stronger (Figure 6). After CBB staining of the culture supernatant at each time point, a clear band for secreted V_HH_-His was confirmed (Figure 6B, inset, and Figure S5B). The concentrations of V_HH_-His were calculated by using a BSA standard curve, and the data indicated its increasing trend over time.

**Figure 7:**
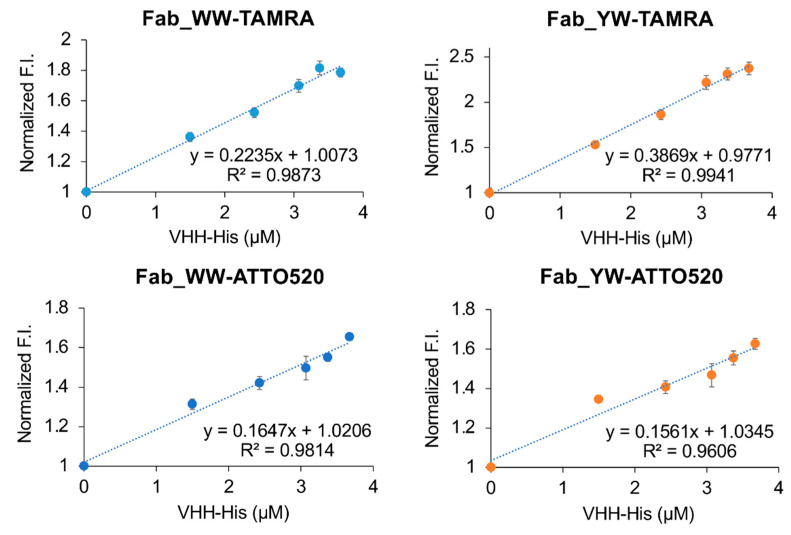
The relationship between the fluorescence intensity displayed by Q-body in 50% M9 culture medium and the concentration determined by a CBB-stained SDS-PAGE gel with known amounts of bovine serum albumin (BSA) standards.

To verify the relationship between the fluorescence signal displayed by the Q-body and the concentration of V_HH_-His in a 50% M9 culture medium, we made correlation charts using these two data sets and four types of Q-bodies (Figure 7). The high correlation coefficient R^2^ (Fab_WW-TAMRA: 0.99; Fab_YW-TAMRA: 1.00; Fab_WW-ATTO520: 0.99; Fab_YW-ATTO520: 0.98) indicated that the fluorescence signal displayed by the Q-body in 50% culture medium has a good positive correlation with the concentration of V_HH_-His. In particular, Fab_YW-TAMRA showed the best correlation. These results proved that the Q-body can detect the target antigen during the bacterial production process of recombinant nanobody.

Finally, the specificity of the Fab variants and the Q-bodies derived of were tested (Figure S7). As a result, the specific recognition of C-terminal His-tag by Fab Q-body variants (WW and YW) was clearly demonstrated by antigen-coated ELISA (Figure S7A,B). Also, TAMRA- and ATTO520-labeled Q-body variants specifically recognized His_6_ peptide among other popular tag peptides (Figure S7C).

### 3.6. Docking Simulation of Antigen to Mutant Antibodies

To better understand the molecular mechanism behind the affinity modulation upon three types of Y to W mutations in CDR_H1_, a docking simulation of the four types of scFv and the ligand (His_4_) were performed using CDOCKER, a molecular dynamics (MD) simulated-annealing-based algorithm [21]. After mutation and energy minimization of each variant, docking simulation was performed. Diverse poses were generated adopting the random rigid body rotation and simulated annealing. To initiate this mechanism, all the default parameters were considered allowing the generation of 10 poses for every ligand. The docking estimation was performed by the -CDOCKER energy, which was calculated, based upon the internal ligand strain energy and receptor–ligand interaction energy. Additionally, -CDOCKER interaction energy signifies the energy of the non-bonded interaction that exists between the protein and the ligand. In both cases, greater -CDOCKER energy and -CDOCKER interaction energy value implies greater favorable binding between the protein and the ligand [25].

**Figure 8:**
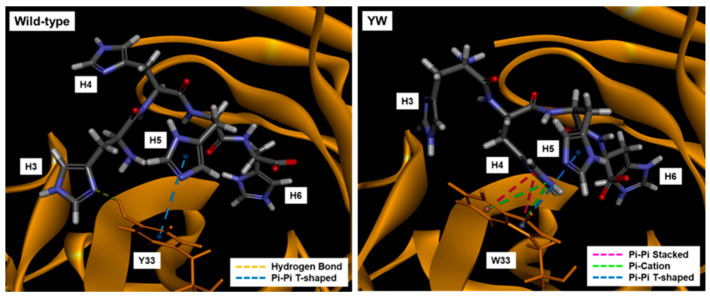
Docking poses of the wild-type and YW mutant.

As a result, the -CDOCKER Energy and -CDOCKER Interaction Energy of the wild-type and the mutants are shown in Table 4. The order of -CDOCKER Energy, which indicates the sum of the intermolecular interaction energy and the internal energy of the ligand molecule, was WW, YW, the wild-type and WY, which was in good agreement with the ligand-binding affinity of scFv and Fab variants by ELISA (Figure 1C) and BLI (Table 1). It is worth noting that -CDOCKER Energy was significantly increased by the Y33W mutation, suggesting that this mutation is particularly important for improving the affinity. To better understand this phenomenon, the docking poses of the wild-type and YW in the binding pocket were compared (Figure 8). Since the indole of the Trp side chain has a bicyclic structure, the mutation may have widened the scope for intermolecular interactions with the imidazole derived from His (H4) including π–π stacking, π–Cation and π–π T-shape, compared with that of Tyr, which probably lead to the improved affinity.

## 4. Conclusions

In this study, we successfully constructed a fluorescent immunosensor Q-body for His-tag detection. Because of its ability to display an antigen-dependent fluorescence signal, it can also be used for the simple quantitative determination of the target antigen. The Q-body assay is simple to operate and can obtain results quickly (the fluorescence signal can be detected immediately after mixing with the antigen), therefore, it can be expected to contribute to the real-time monitoring of target recombinant products both in academic research and in practical industrial bioprocessing.

To demonstrate the merit of our method more clearly, a table that compares the His-tag detection methods reported to date was compiled (Table 5). The comparison therein clearly shows the comparative detection range and the merits of the Q-body assay, especially in the time and the number of reagents required.

In addition, by introducing a mutation into Tyr33 in the 3D5 antibody’s V_H_ region to increase the Trp contents, not only was improvement of the quenching effect obtained, but also higher affinity to the His_6_ ligand, resulting in the increased antigen detection sensitivity of the Fab-type Q-body. This approach could provide many useful ideas for designing other effective Q-bodies in the future.

## Figures and Tables

**Table 1:** Kinetic parameters of 3D5 antibody fragments and their Q-bodies with biotinylated His_6_ peptide. An average and a standard error from global fitting are shown (n = 3).

Protein	ka (×10^3^ M^−1^s^−1^)	kd (×10^−5^ s^−1^)	K_D_ (× 10^−9^ M)
scFv	84.5 ± 0.9	42.5 ± 1.7	5.03 ± 0.21
scFv_WW	290 ± 2	83.5 ± 1.5	2.88 ± 0.06
scFv_TAMRA Q-body	113 ± 1	303 ± 2	26.8 ± 0.3
scFv_WW_TAMRA Q-body	* nd	* nd	* nd
Fab	27.4 ± 0.3	9.93 ± 0.38	3.62 ± 0.14
Fab_WW	132 ± 0.7	2.27 ± 0.60	0.17 ± 0.05
Fab_YW	139 ± 0.7	10.5 ± 0.8	0.76 ± 0.06
Fab_TAMRA Q-body	210 ± 1.4	67.2 ± 1.9	3.19 ± 0.09
Fab_WW_TAMRA Q-body	264 ± 1.8	45.5 ± 1.3	1.72 ± 0.05
Fab_YW_TAMRA Q-body	392 ± 3.8	51.2 ± 2.8	1.31 ± 0.07

Data sets with *X*^2^ < 0.4, *R*^2^ > 0.96 are used. * nd: not detected.

**Table 2:** EC50 and LOD of Fab-type Q-bodies to His_6_ peptide.

Fab Q-Body and Dye Used	EC50 (μM)	LOD (μM)
Wild-type TAMRA	132 ± 34	3.5
WW TAMRA	89.5 ± 25.4	2.7
YW TAMRA	54.7 ± 9.5	3.1
Wild-type ATTO520	13.8 ± 5.1	1.0
WW ATTO520	8.8 ± 1.3	0.3
YW ATTO520	6.1 ± 1.4	0.2

**Table 3:** Sensitivity of Fab-type Q-bodies to His-tagged protein in 50% culture medium.

Fab Q-Body and V_HH_-His	EC50 (μM)	LOD (µM)
WW-TAMRA	11.5 ± 0.4	0.37
YW-TAMRA	11.1 ± 2.4	0.57
WW-ATTO520	10.7 ± 4.6	0.035
YW-ATTO520	3.0 ± 0.4	0.0095

**Table 4:** -CDOCKER Energy and -CDOCKER Interaction Energy calculated by CDOCKER in Discovery Studio for each mutant.

	- CDOCKER Energy (kcal/mol)	- CDOCKER Interaction Energy (kcal/mol)
**Wild-type**	81.5	61.6
**YW**	90.9	63.1
**WY**	78.9	58.9
**WW**	93.7	70.9

**Table 5:** Comparison of the reported methods to detect His-tagged proteins.

Method	Detection Range	Time Required	Steps	Number of Reagents
His-Tag Protein Expression Check Kit (Abcam, Cambridge, UK)	0.01–0.5 mg/mL (0.2~10 µM *)	10~15 min	1	One LF strip + running buffer
His Tag ELISA Detection Kit (GenScript, Piscataway, NJ, USA)	1~729 ng/mL (0.02~14.6 nM *)	1.5 h	7	Many
InVision His-tag In-gel Stain (Invitrogen, Thermo-Fisher)	>0.5 pmole in SDS-PAGE gel (>50 nM in 10 µL)	>70 min	5	3
Ni-NTA-Atto Conjugates (Spelco, Sigma-Aldrich)	>50 ng in SDS-PAGE gel (> 0.1 µM * in 10 µL)	4~18 h	5	>2
CBGlyco™ 6xHis Protein Tag Stain Kit (Creative Biolabs, Shirley, NY, USA)	>0.2 µg in SDS-PAGE gel (0.4 µM * in 10 µL)	2 h	5	2
Pro-Detect Rapid assays (Thermo Fisher Scientific)	4~20 µg/mL (0.1~0.4 µM *)	10~15 min	1	One LF strip + running buffer
Competitive FRET immunoassay [8]	>0.8 µM	90 s	1	2 (fluorolabeled Ab + quencher-labeled peptide)
This work	>0.2 µM	<60 s	1	1

* calculated assuming that the molecular weight of target protein is 50 kD.

## Supplementary Materials

The following are available online at https://www.mdpi.com/article/10.3390/s21154993/s1, Table S1: List of primers used in this study; Table S2: Positions of Trp residues in the wild-type and mutant VH regions; Figure S1: Preparation of biotinylated His6 peptide by Fmoc solid-phase peptide synthesis; Figure S2: 3D models of 3D5 antibody variants; Figure S3: CBB-stained SDS-PAGE for scFv/Fab 3D5 and their variants; Figure S4: Fluorescence quenching and recovery of scFv Q-body variants; Figure S5: BLI sensorgrams; Figure S6: CBB-stained SDS-PAGE of VHH-expressing recombinant *Brevibacillus* culture supernatant; Figure S7: Specificity of Fab variants and their Q-bodies.
