# Supplementary material for: Construction of Fluorescent Immunosensor Quenchbody to Detect His-Tagged Recombinant Proteins Produced in Bioprocess
Source: Sensors (Basel). 2021 Jul 22;21(15):4993. doi: 10.3390/s21154993 (PMC8347774; doi:10.3390/s21154993)
Supplement: Supplementary file 1 [file sensors-21-04993-s001.zip › sensors-1298323-supplementary.pdf]

Supplementary Materials for

Construction of fluorescent immunosensor Quenchbody for detecting His-tagged recombinant proteins produced in bioprocess

Xuerao Ning[1], Takanobu Yasuda[1], Tetsuya Kitaguchi[2], Hiroshi Ueda[2*]

[1] Graduate School of Life Science and Technology, Tokyo Institute of Technology, 4259-R1-18 Nagatsuta-cho, Midori-ku, Yokohama, Kanagawa 226-8503, Japan

[2] Laboratory for Chemistry and Life Science, Institute of Innovative Research, Tokyo Institute of Technology, 4259-R1-18 Nagatsuta-cho, Midori-ku, Yokohama, Kanagawa 226-8503, Japan

Corresponding author: ueda@res.titech.ac.jp

Table of Contents

Table S1. List of primers used in this study. S-2

Table S2. Positions of Trp residues in the wild-type and mutant V_H regions. S-2

Figure S1. Preparation of biotinylated His_6 peptide by Fmoc solid-phase peptide synthesis. S-3

Figure S2. 3D models of 3D5 antibody variants. S-5

Figure S3: CBB-stained SDS-PAGE for scFv/Fab 3D5 and their variants. S-5

Figure S4. Fluorescence quenching and recovery of scFv Q-body variants. S-6

Figure S5. BLI sensorgrams for the Fab fragments used for making Table 1. S-7

Figure S6. CBB-stained SDS-PAGE of V_{HH}-expressing recombinant *Brevibacillus* culture supernatant. S-8

Figure S7. Specificity of Fab variants and their Q-bodies S-9

Table S1. List of primers (5´-3´) used in this study. Restriction sites are underlined, while mutated bases are shown in small letters.

3D5_Age_back	CTCTAATGAGACCGGTGGAGG
3D5_Sal_for	GTGGTGGTGGTCGACCTTGATTTCAAGCTTCGTGC
In-fusion_3D5_VH_for	TGGTGGAAGCGCTCGAGACGGTCACCGTGGTG
In-fusion3D5VLback	CTCTAATGAGACTAGTGACATTCTCATGACTCAGACC
In-fusion3D5VLfor	GTTTGATTTCAAGCTTCGTGCCTG
3D5VH_WY_back	CTGGTTATACTTTCACCGACTtgTACATGAATTGG
3D5VH_WW_back	CTGGTTATACTTTCACCGACTggTggATGAATTGGG
3D5VH_YW_back	CTGGTTATACTTTCACCGACTATTggATGAATTGGG
3D5VH31For	AGTCGGTGAAAGTATAACCAGAC
pBIC_pET1-4VHH_Rv	CATCCTGTTAAGCTTTGTTAGCAGCCGGATC
pBIC3-forwardVHH	AGTTCCGCATTCGCTCAGGTGCAGCTCGTG

Table S2. Positions of Trp residues in the wild-type and mutant V regions. The residue numbers are according to Kabat numbering scheme.

	CDRH1										
Kabat No.	H32	H33	H34	H35	H36	...	H47	...	H103	...	L35
scFv/Fab	Y	Y	M	N	**W**		**W**		**W**		**W**
scFv/Fab_WW	**W**	**W**	M	N	**W**		**W**		**W**		**W**
scFv/Fab_WY	**W**	Y	M	N	**W**		**W**		**W**		**W**
scFv/Fab_YW	Y	**W**	M	N	**W**		**W**		**W**		**W**

A

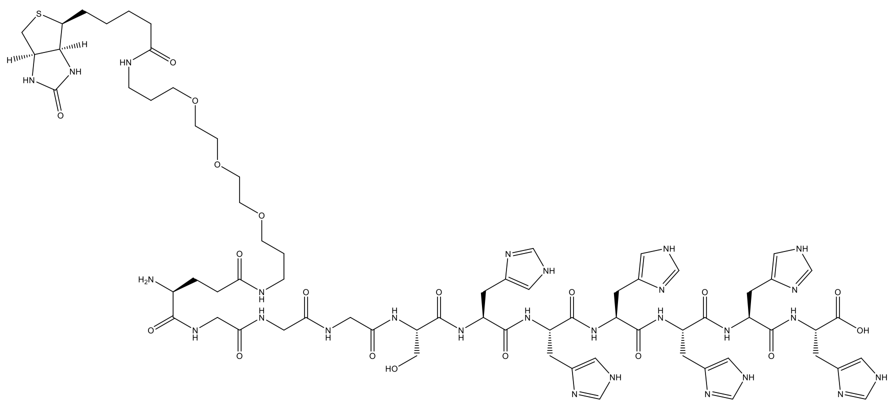

Glu (biotinyl-PEG)-Gly-Gly-Gly-Ser-His-His-His-His-His-His-COOH

Chemical Formula: $C_{70}H_{101}N_{27}O_{19}S$

Exact Mass: 1655.75

Molecular Weight: 1656.81

m/z: 1655.75 (100.0%), 1656.75 (87.2%), 1657.76 (29.7%), 1657.75 (12.3%), 1658.76 (10.6%), 1658.75 (7.5%), 1657.74 (5.0%), 1659.76 (3.4%), 1659.75 (2.0%), 1656.76 (1.2%)

Elemental Analysis: C, 50.75; H, 6.14; N, 22.83; O, 18.35; S, 1.94

B

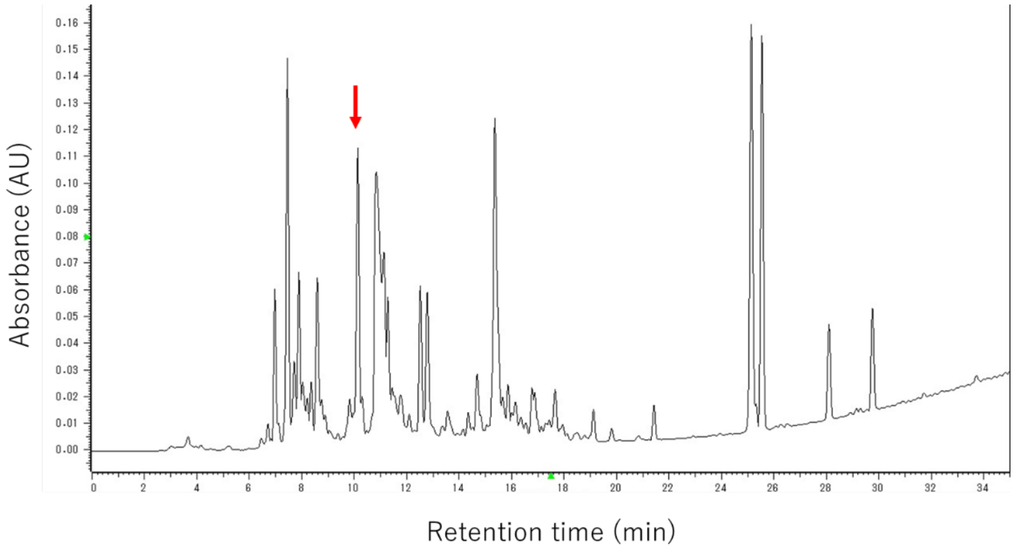

C

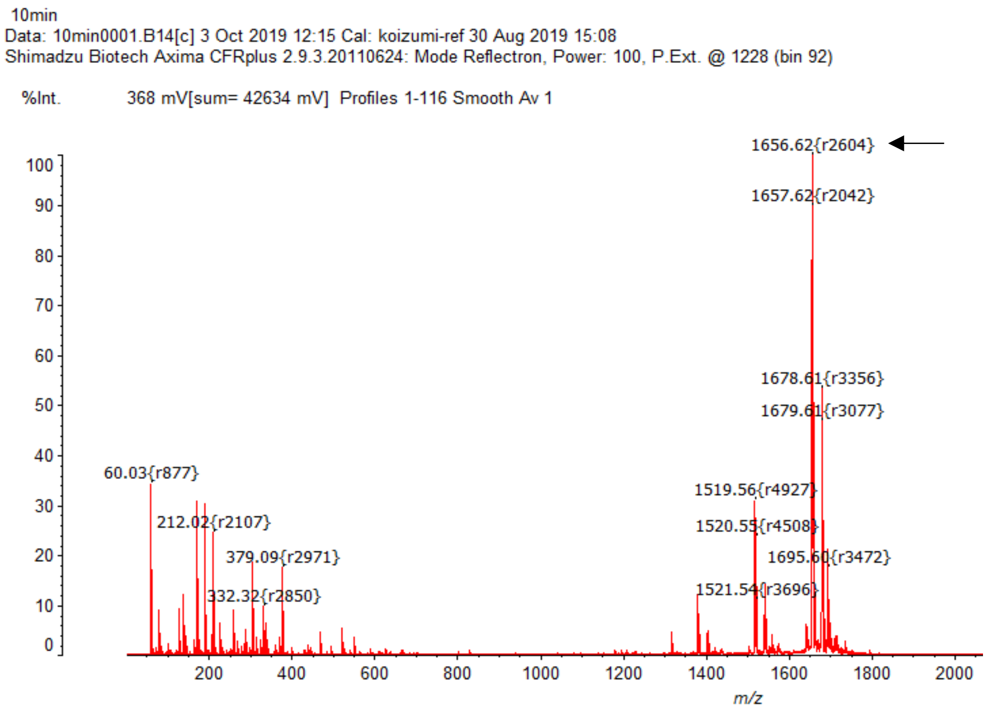

Figure S1: Preparation of biotinylated His_6 peptide by Fmoc solid-phase peptide synthesis. (A) Structural formula drawn with ChemDraw 19.0. (B) Reversed phase high performance liquid chromatography (RP-HPLC) of the sample obtained by Fmoc peptide synthesis. Column: $5C_{18}$-AR-II (4.6ID × 250mm). Gradient A (H_2O): 100% (0 min) – 0% (35min), Gradient B (CH_3CN): 0% (0 min) – 100% (35min). Flow rate: 1 mL/min. (C) MALDI-TOF MS spectrum. Biotinylated His_6 was detected at m/z 1656.62.

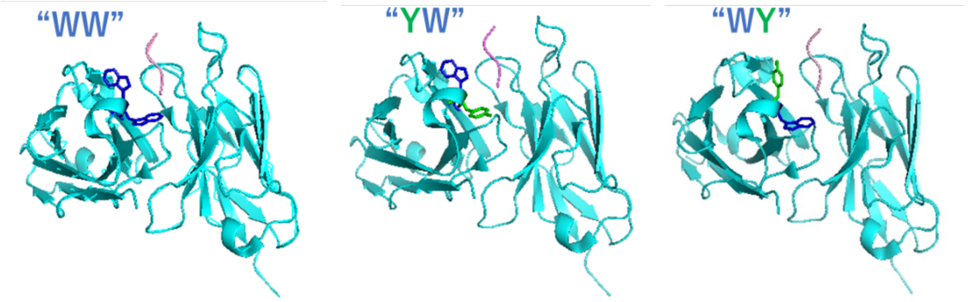

Figure S2. 3D models of 3D5 antibody variants based on the 3D5 V_L-V_H scFv (PDB ID: 1KTR). Left: heavy chain. Right: Light chain. Blue: mutated positions (H32 and H33, according to the Kabat numbering scheme). Magenta: His-tag (4-6). The rotamers of mutated amino acid residues with the highest probability are calculated and shown by UCSD Chimera ("WW" : HY32W + HY33W; "YW": HY33W; "WY": HY32W).

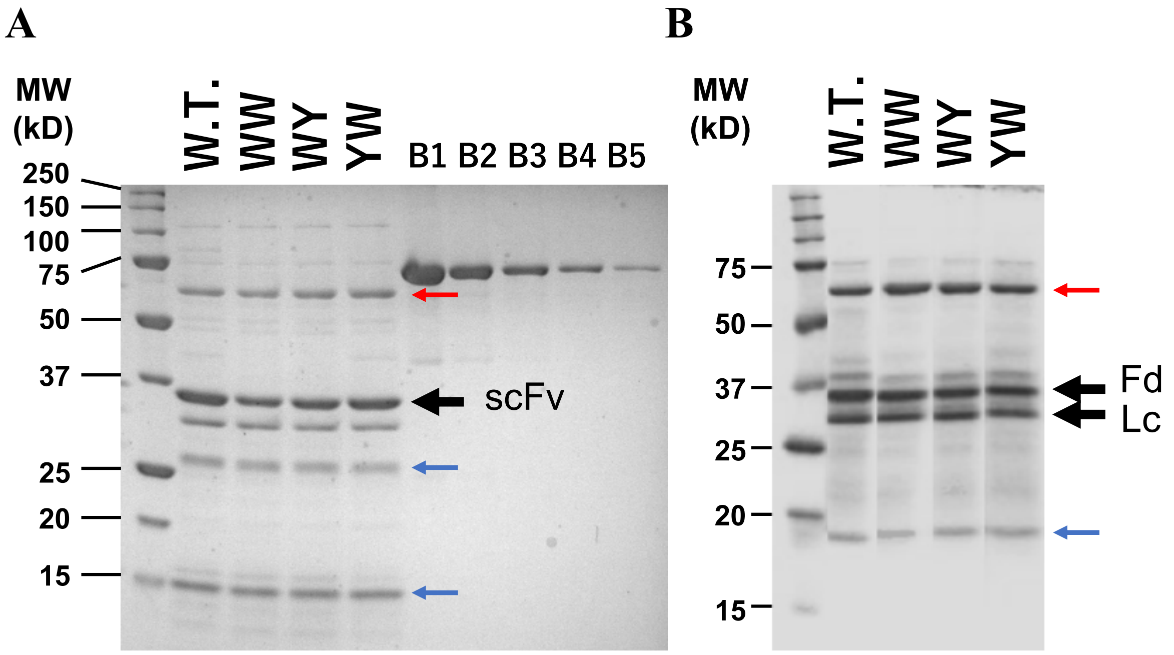

Figure S3. CBB-stained SDS-PAGE for unlabeled scFv/Fab 3D5 and their variants. (A) scFv 3D5 and (B) Fab 3D5. The positions of the scFv, heavy (Fd=V_H-C_H1) and light (Lc) chains are shown with bold arrows, while the positions for GroEL are shown with red arrows. The faint smaller bands shown with blue arrows may represent His-tagged degradation products containing the V_L or C_H1 domain in A and B, respectively, because they are different in the two panels. The concentration of each protein was calculated by quantifying the band density of scFv (A) or L chain (B) by using the BSA standards shown in the lanes B1~B5: 1000, 500, 250, 125, and 62.5 ng of BSA.

A

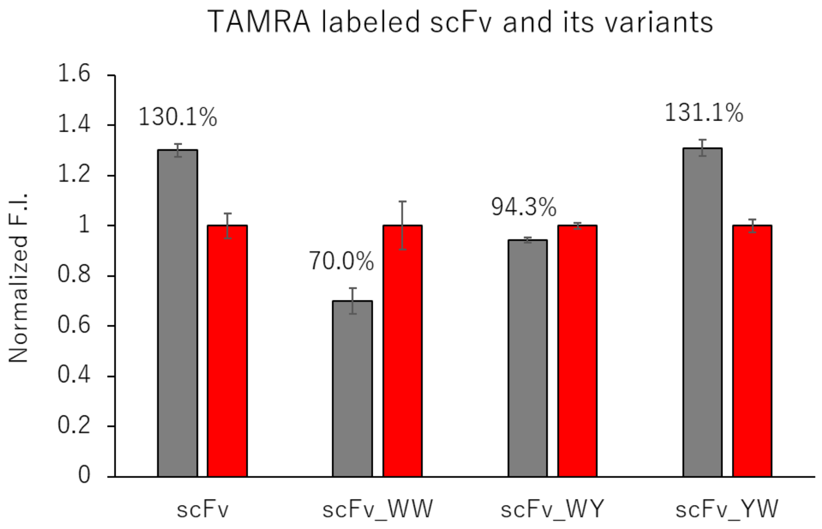

B

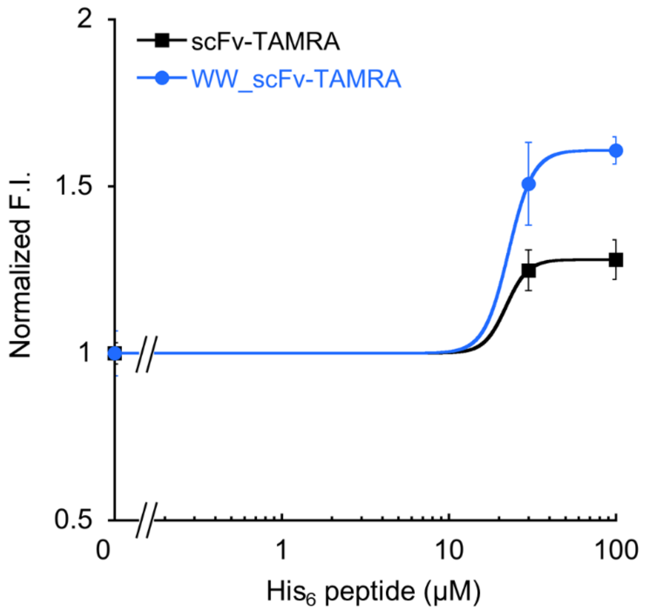

Figure S4. Fluorescence quenching and recovery of 5-TAMRA C6 labeled scFv 3D5 Q-body variants. (A) Gray and red bars represent the fluorescence intensity of non-denatured and denatured Q-bodies, respectively. The fluorescence intensity of each Q-body in PBST was normalized by the mean intensity of 1 nM Q-body in the denaturant (7 M Guanidium hydrochloride, 100 mM DTT). Error bars indicate ±1 standard deviation (SD) (n = 3). (B) His_6 peptide dose-response of 5-TAMRA C6-labeled wild-type and WW-mutant scFv Q-bodies in PBST. Error bars indicate ±1 SD (n = 3).

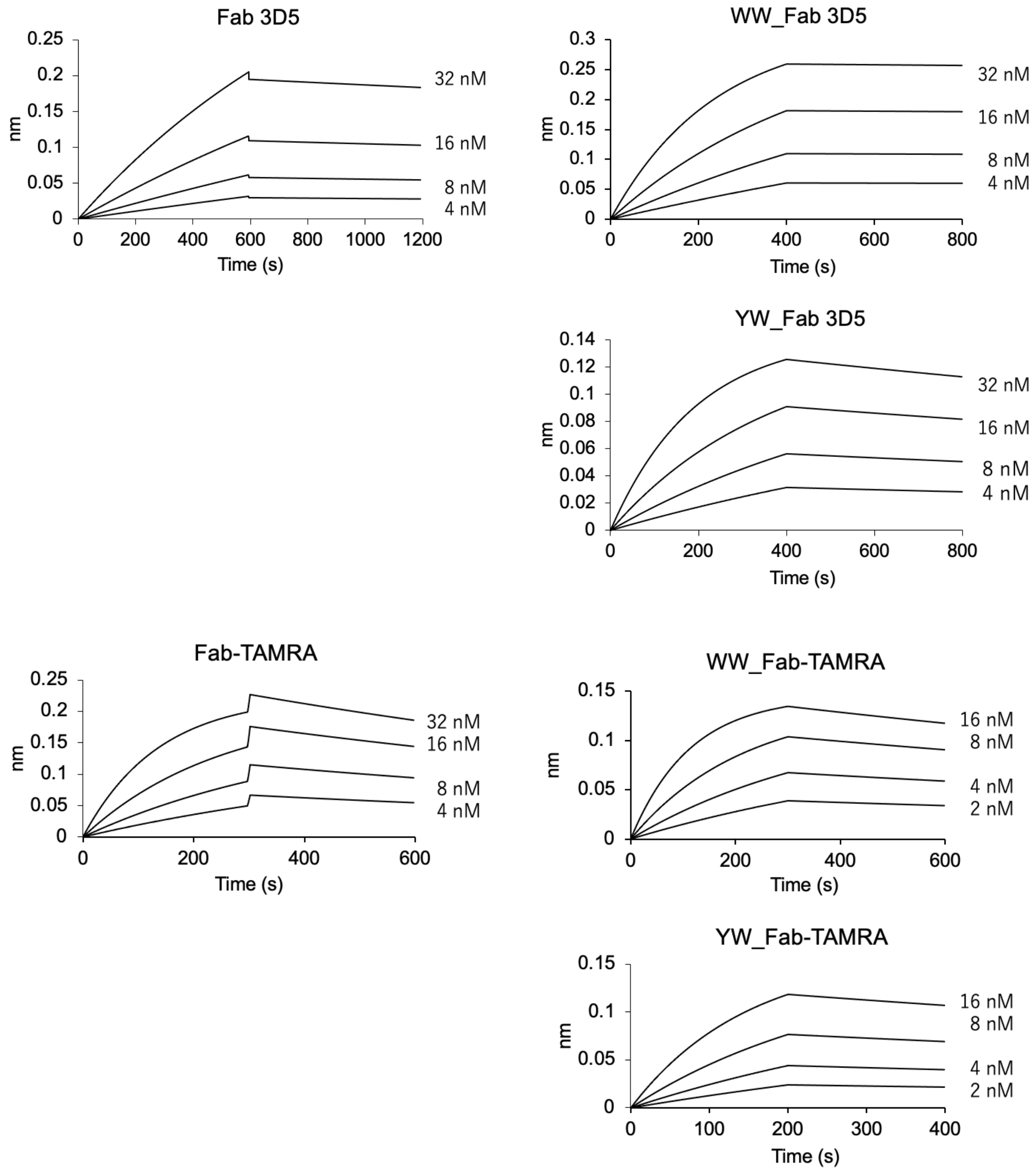

Figure S5. BLI Sensorgrams for the Fab fragments used to derive kinetic constants shown in Table 1.

A

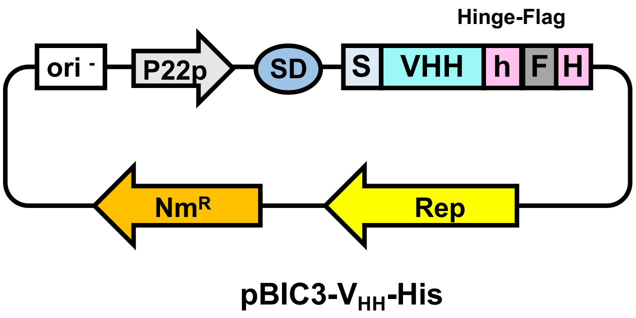

B

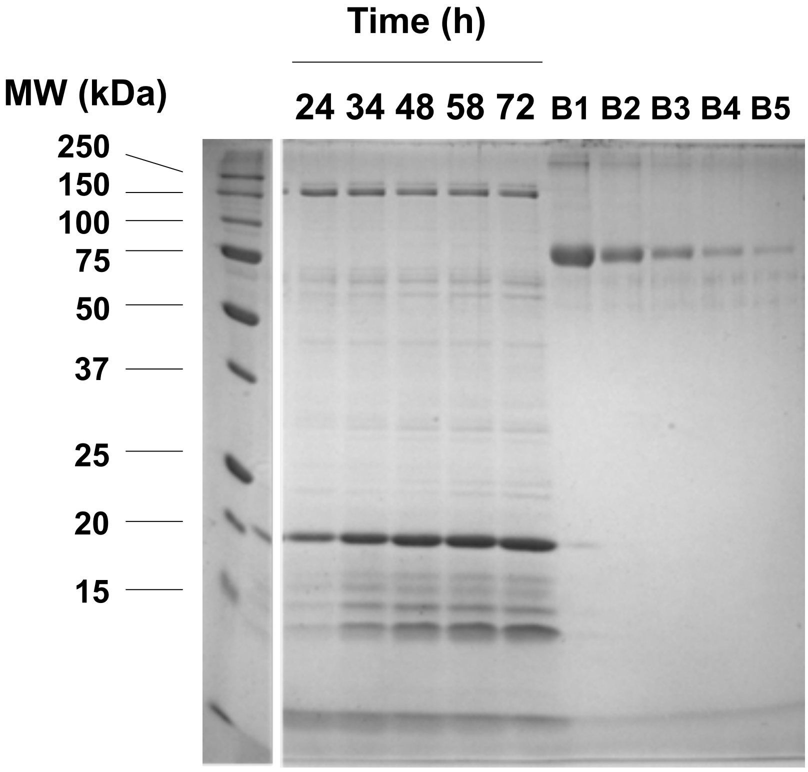

Figure S6. CBB-stained SDS-PAGE of recombinant *Brevibacillus* culture supernatant. (A) Schematic structure of V_{HH}-His expression vector. (B) CBB-stained SDS-PAGE of the culture supernatant including V_{HH}-His (21.7 kDa) (9 μL) after the culture in M9Nm medium for the indicated periods. The concentration of secreted V_{HH}-His was analyzed using BSA standards. B1-B5: 1000 ng, 500 ng, 250 ng, 125 ng, and 62.5 ng of BSA.

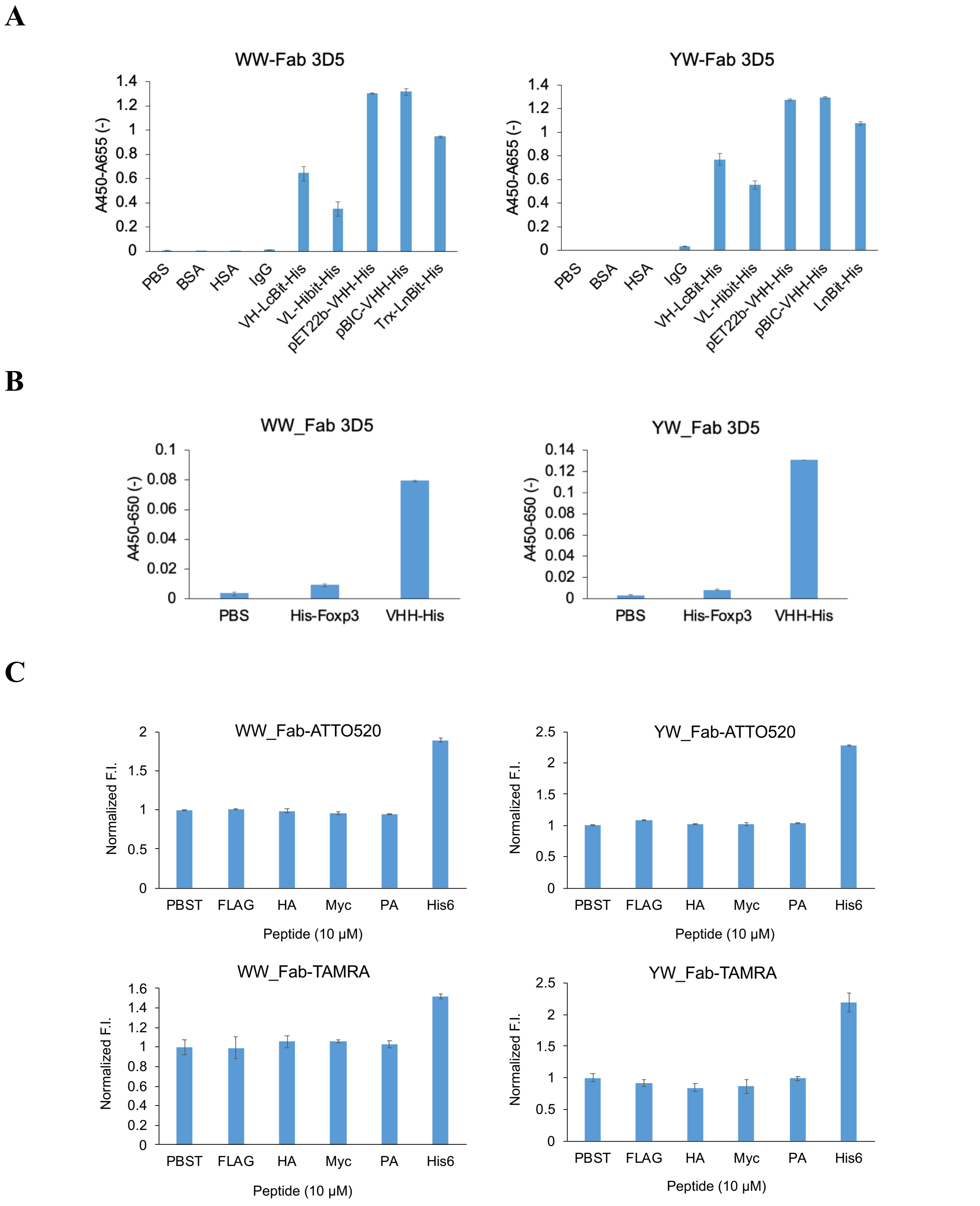

Figure S7. Specificity of the Fab variants and their Q-bodies to C-terminal His-tagged protein and His_6 peptide, respectively. (A) The indicated proteins (2 μg/mL) were immobilized and probed with indicated Fab (2 μg/mL), which was detected by anti-Flag M2 horseradish peroxidase (HRP, Sigma). (B) The indicated immobilized proteins (2 μg/mL) were probed with indicated Fab (2 μg/mL) and anti-myc HRP conjugate (Genscript). (C) The indicated Q-body (1 nM) was mixed with indicated peptides (10 μM) and measured for the fluorescence. VH-LcBiT-His, VL-HiBiT-His and Trx-LnBiT-His was prepared as described in [19].
